# Sex ratio influences the motivational salience of facial attractiveness

**DOI:** 10.1098/rsbl.2014.0148

**Published:** 2014-06

**Authors:** Amanda C. Hahn, Claire I. Fisher, Lisa M. DeBruine, Benedict C. Jones

**Affiliations:** Institute of Neuroscience and Psychology, University of Glasgow, Glasgow, UK

**Keywords:** incentive salience, operational sex ratio, intrasexual competition, beauty, mate preferences

## Abstract

The sex ratio of the local population influences mating-related behaviours in many species. Recent experiments show that male-biased sex ratios increase the amount of financial resources men will invest in potential mates, suggesting that sex ratios influence allocation of mating effort in humans. To investigate this issue further, we tested for effects of cues to the sex ratio of the local population on the motivational salience of attractiveness in own-sex and opposite-sex faces. We did this using an effort-based key-press task, in which the motivational salience of facial attractiveness was assessed in samples of faces in which the ratio of male to female images was manipulated. The motivational salience of attractive opposite-sex, but not own-sex, faces was greater in the own-sex-biased (high competition for mates) than in the opposite-sex-biased (low competition for mates) condition. Moreover, this effect was not modulated by participant sex. These results present new evidence that sex ratio influences human mating-related behaviours. They also present the first evidence that the perceived sex ratio of the local population may modulate allocation of mating effort in women, as well as men.

## 1. Introduction

The sex ratio of the local population (i.e. ratio of males to females) influences mating-related behaviours in many species. For example, in many non-human species, greater selectivity is evident in females’ mate preferences when the local population's sex ratio is male biased than when it is female biased (reviewed in [1]). Additionally, increasing the proportion of competitors for mates intensifies intrasexual competition (reviewed in [1]). Possible effects of the sex ratio of the local population on human mating-related behaviours have also been reported. For example, women in geographical regions with higher proportions of men show greater selectivity in their mate choices [2], while regions with higher proportions of women have a greater prevalence of both polygyny [3] and short-term mating strategies [4].

More recent work suggests that the sex ratio of the local population may also influence how men allocate mating effort [5]. For example, cues that the sex ratio of the local population is male biased increase (i) men's willingness to incur financial debt to obtain immediate resources and (ii) the amount of financial resources people believe men should invest in potential mates. While these results suggest that male-biased sex ratios increase the amount of *financial* resources men will invest in potential mates, it is not known whether this pattern of results also occurs for other measures of mating effort.

In light of the above, we investigated the effects of manipulating cues to the sex ratio of the local population on the motivational salience of attractiveness in own-sex and opposite-sex faces. We measured the motivational salience of attractive faces using a standard key-press (i.e. effort-based) task in which participants can control the viewing duration for faces [6,7]. Given men appear to invest more financial resources in potential mates when perceived competition for mates is intense [5], we predicted that the motivational salience of attractive opposite-sex, but not own-sex, faces would be greater in our own-sex-biased condition than in our opposite-sex-biased condition.

## 2. Material and methods

### (a) Stimuli

In an initial pilot study, 100 heterosexual men and 100 heterosexual women (mean age = 24.67 years, s.d. = 5.87 years) rated the attractiveness of 50 young white men's faces (mean age = 24.24 years, s.d. = 3.99 years). A different group of 100 heterosexual men and 100 heterosexual women (mean age = 24.98 years, s.d. = 5.56 years) rated the attractiveness of 50 young white women's faces (mean age = 24.26 years, s.d. = 4.01 years). All faces had direct gaze and neutral expressions and the photographs were taken under standardized conditions. The order in which the faces were presented for rating was fully randomized and ratings were made using 1 (much less attractive than average) to 7 (much more attractive than average) scales. Male and female faces were presented in separate blocks of trials. Inter-rater agreement for ratings of the male and female faces was high (both Cronbach's *α* > 0.96), and men's and women's ratings were highly correlated for both male and female faces (both *r* > 0.97).

The average attractiveness ratings of the individual faces in the two sets were used to identify the eight most attractive male faces (*M* = 3.54, s.d. = 0.27) and the eight most attractive female faces (*M* = 3.79, s.d. = 0.35). These 16 images are referred to hereon as the *high attractiveness targets*. The average attractiveness ratings were also used to identify the eight least attractive male faces (*M* = 1.69, s.d. = 0.19) and the eight least attractive female faces (*M* = 1.79, s.d. = 0.29) in the two sets. These 16 images are referred to hereon as the *low attractiveness targets*. The high and low attractiveness targets were used to assess the motivational salience of relatively attractive and relatively unattractive faces in the motivational salience test. We also used these ratings to identify the 16 male faces (*M* = 2.63, s.d. = 0.21) and 16 female faces (*M* = 2.67, s.d. = 0.24) around the median in the male and female face sets. These 32 images are referred to hereon as the *filler faces* and were used to manipulate the sex ratio of the sample of images presented in the motivational salience test.

### (b) Procedure

Two hundred and ninety-one heterosexual men and 292 heterosexual women (mean age = 24.25 years, s.d. = 5.93 years) participated in the main online experiment. Participants were recruited by following links on social bookmarking websites (e.g. stumbleupon.com), participated remotely (i.e. not in the presence of an experimenter) and received no compensation for participating. The website where the experiment was run required that participants register with a unique username prior to participation. No participants took part in both the pilot study and main experiment. Each participant completed a ‘pay-per-view’ key-press task, similar to those that have been used in previous studies to assess the motivational salience of attractive faces [6,7].

In the key-press tasks, participants can control the viewing duration of the face images presented by repeatedly pressing designated keys on their keyboard after initiating each trial by pressing the space bar. Here, participants could either increase the length of time a given face was displayed by alternately pressing the 7 and 8 keys or decrease the length of time a given face was displayed by alternately pressing the 1 and 2 keys. Each key press increased or decreased the viewing duration by 100 ms. The default viewing duration for each image (i.e. the length of time a face remained onscreen if no keys were pressed) was 4 s. Participants were told that the key-press task would last for a total of 3 min in order to discourage responses aimed at changing the length of engagement with the task. However, in reality, the total length of the key-press task was dependent on participants’ responses. All participants key-pressed at least once during the experiment. All participants completed a brief training task designed to familiarize them with the key-press procedure prior to beginning the experiment. Faces were not presented in this training task.

Participants were randomly allocated to one of three versions of the key-press task: a male-biased sex ratio version, a female-biased sex ratio version or an unbiased sex ratio version. The 16 high attractiveness targets and 16 low attractiveness targets were presented in each version of the task. In addition to these target faces, however, the male-biased sex ratio version also included the 16 male filler faces, the female-biased sex ratio version also included the 16 female filler faces, and the unbiased sex ratio version also included the eight male and eight female filler faces around the median attractiveness rating for the male and female face sets. In each version of the task, all faces were presented in a single block of trials in which trial order was fully randomized. Random allocation of participants to versions was done separately for male and female participants to ensure task version was not confounded with participant sex.

### (c) Initial processing of data

First, we calculated the key-press score for each of the *target* faces, separately for each participant. Following previous work [6,7], key-press scores for each face were calculated by subtracting the total number of key presses that decreased viewing duration from the total number of key presses that increased viewing duration. Faces with greater key-press scores are then those that the participant was willing to expend more effort to view. For each participant, we then calculated their *attractiveness motivation score* for male faces (the extent to which they expended more effort to view male high attractiveness targets than male low attractiveness targets) by subtracting their mean key-press score for the male low attractiveness targets from their mean key-press score for the male high attractiveness targets. A corresponding *attractiveness motivation score* for female faces was also calculated for each participant. One-sample *t*-tests showed that both men's and women's attractiveness motivation scores were significantly greater than chance (i.e. 0) for both male and female faces (all *p* < 0.001).

## 3. Results

Attractiveness motivation scores were analysed using a mixed-design ANOVA with *sex of target face* (opposite-sex and own-sex) as a within subject factor and *task version* (opposite-sex bias, own-sex bias and unbiased) and *participant sex* (male and female) as between subjects factors. Note that *sex of target face* and *task version* are both coded relative to each participant's sex, allowing us to directly test whether the predicted effects involving *sex of target face* and *task version* are significantly different for male and female participants. The data set is available as the electronic supplementary material.

Our analysis revealed a significant main effect of *sex of target face* (*F*_1,577_ = 197.73, *p* < 0.001, 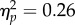), whereby attractiveness motivation scores were greater for opposite-sex faces (*M* = 16.86, s.e.m. = 0.74) than own-sex faces (*M* = 7.20, s.e.m. = 0.41). The interaction between *sex of target face* and *participant sex* was also significant (*F*_1,577_ = 56.79, *p* < 0.001, 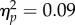); the effect of *sex of target face* on attractiveness motivation scores was significantly greater for male participants *(M* = 14.83, s.e.m. = 1.17) than female participants (*M* = 4.48, s.e.m. = 0.72).

**Figure 1.**
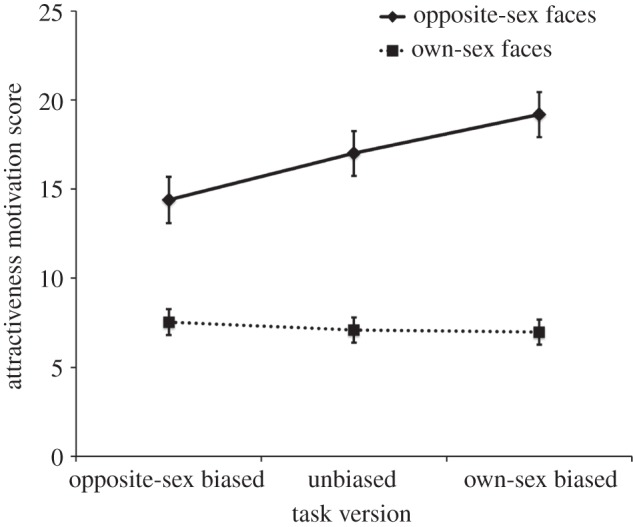
The significant two-way interaction between *sex of target face* and *task version*. Means and s.e.m. are shown.

As we had predicted, the interaction between *sex of target face* and *task version* was also significant (*F*_2,577_ = 5.05, *p* = 0.007, 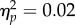, figure 1). No other effects, including the three-way interaction among *sex of target face*, *task version* and *participant sex*, were significant (all *F* < 1.56, all *p* > 0.21, all 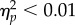).

To interpret the two-way interaction between *sex of target face* and *task version*, we first tested for linear effects of task version on attractiveness motivation scores for opposite-sex and own-sex faces, with the opposite-sex-biased task version coded as 1, the unbiased task version coded as 2 and the own-sex-biased task version coded as 3. These analyses revealed a significant linear effect of *task version* for opposite-sex faces (*p* = 0.010), but not own-sex faces (*p* = 0.65). Additionally, independent samples *t*-tests showed that attractiveness motivation scores for opposite-sex faces differed significantly between the own-sex- and opposite-sex-biased task versions (*p* = 0.012), but not between the own-sex-biased and -unbiased task versions (*p* = 0.28) or between the opposite-sex-biased and -unbiased task versions (*p* = 0.11). Repeating these comparisons for own-sex faces revealed no significant differences (all *p* > 0.66).

## 4. Discussion

Consistent with previous research [6,7], attractiveness motivation scores were greater for opposite-sex than own-sex faces. We also found that attractiveness motivation scores for opposite-sex faces were greater in the own-sex-biased (high competition for mates) than opposite-sex-biased (low competition for mates) condition. Condition had no effect on attractiveness motivation scores for own-sex faces, however. Given that the motivational salience of attractive opposite-sex faces is likely to be a proxy for willingness to allocate mating effort to attractive potential mates [6], our results complement recent work suggesting that own-sex-biased sex ratios increase the amount of financial resources men invest in potential mates [5]. Increasing the perceived intensity of competition for mates by manipulating cues to the sex ratio of the local population decreases, rather than increases, sensitivity to attractive traits in potential mates [1]. Consequently, our results are unlikely to simply reflect the effects of changes in participants’ sensitivity to attractiveness on key-press task responses.

Although recent work found that own-sex-biased sex ratios increased the amount of financial resources men invested in potential mates, sex ratios did not influence how women allocated financial resources [5]. By contrast, no sex difference in the effect of sex ratio on the motivational salience of attractive faces was observed in the current experiment. We suggest that this difference occurred because financial resources are more important for men's than women's mate value while both men and women value attractiveness in potential mates [8].

Previous research on the motivational salience of facial attractiveness has emphasized how much effort individuals allocate, on average, to attractive and unattractive faces [6,7]. By contrast, our data demonstrate that individuals allocate effort to attractive and unattractive individuals facultatively, changing response patterns according to perceived characteristics of the local population. More fundamentally, our data present new evidence that cues to the sex ratio of the local population can directly influence mating-related behaviours in humans, complementing research on mating-related behaviours in other species.

## Supplementary Material

RSBL-2014-0148_supp

## Notes

The study was approved by the local ethics committee.
